# Latent Profiles of Burnout, Self-Esteem and Depressive Symptomatology among Teachers

**DOI:** 10.3390/ijerph17186760

**Published:** 2020-09-16

**Authors:** Inmaculada Méndez, Juan Pedro Martínez-Ramón, Cecilia Ruiz-Esteban, José Manuel García-Fernández

**Affiliations:** 1 Department of Evolutionary Developmental and Educational Psychology, University of Murcia, 30100 Murcia, Spain; 2 Department of Development Psychology and Didactics, University of Alicante, 03080 Alicante, Spain; josemagf@ua.es

**Keywords:** burnout, quality education, public health, teachers, prevention

## Abstract

Burnout is a reality in the teaching profession. Specifically, teaching staff usually have higher burnout rates. The present study aims to analyze the different burnout profiles and to verify if there were differences between burnout profiles in depressive symptomatology and in the self-esteem of the teachers at school. The total number of participants was 210 teachers from 30 to 65 years. The first scale was the Maslach burnout inventory, the second scale was the Self-Rating depression scale and the third scale was the Rosenberg Self-Esteem Scale. The latent class analysis identified three burnout profiles: the first group with a high level of emotional exhaustion, low personal accomplishment and depersonalization (high burnout); the second group with low emotional exhaustion, low depersonalization and high personal accomplishment (low burnout) and the third group with low depersonalization, low emotional exhaustion and low personal accomplishment (moderate burnout). The results revealed that there were differences in depressive symptomatology (group 1 obtained higher scores than group 2 and group 3) and self-esteem (group 2 obtained higher scores than group 1). The psychological balance and health of teachers depend on preventing the factors that have been associated with this syndrome.

## 1. Introduction

Burnout syndrome has been recognized by the WHO [1], in its International Classification of Diseases (ICD-11), as a syndrome related to chronic stress problems caused by work when these problems have not been successfully managed. It is a three-dimensional syndrome that has the following characteristics: emotional exhaustion—EE (the feeling of exhaustion or lack of energy that can manifest itself physically, psychologically or both); depersonalization—DE (negative or cynical feelings with respect to work) and low personal accomplishment—PA (reduced professional effectiveness, as well as the avoidance of personal and professional relationships) [1,2]. However, it is noteworthy that, in nine of 23 European countries, burnout is not considered a professional illness yet. In fact, it is only possible to recognize exhaustion as an occupational disease in 39% of countries [3].

Researchers have shown that burnout has several consequences for the welfare and health of workers, including teachers. The psychological and physical problems of burnout involve healthcare expenses coupled with costs for companies (high turnover, loss of the most capable talent, low productivity, etc.). It is necessary to assume that large workloads, job insecurity and frustrating routines (e.g., too many meetings) lead to the development of burnout in the workplace [4]. Likewise, according to a systematic review conducted with a wide variety of organizations and companies around the world, including teachers, the level of satisfaction is an important factor that influences the mental and physical health of workers. Workers with low job satisfaction are more vulnerable to experience burnout, anxiety levels, depression and low self-esteem [5].

Specifically, teaching staff usually have higher burnout rates [6,7,8,9,10,11,12,13,14,15,16,17,18,19], especially new teachers [14]. It has been shown that it usually occurs at different educational levels [9,10] and in special education [7]. The existence of medium levels of psychological symptoms has been evidenced, being medium-high for physical and social symptoms [8]. Burnout threatens the physical and mental well-being, self-esteem and the personality of teachers. Stress at work generates demotivation, negative feelings, dissatisfaction, and more besides [15,16], extremely low levels of efficiency, optimism and endurance [9], which can lead to consequences even in students, such as low motivation, school satisfaction and the teacher’s level of perceived care [17]. Emotional exhaustion has been associated with overload, interpersonal conflicts, less social support, less autonomy and less job satisfaction [9,18,19]. Thus, less-committed teachers at work had a higher level of burnout [12].

Several studies on systematic reviews have reported an association between burnout syndrome and depressive symptoms, including teachers, especially with the Maslach burnout inventory (MBI) test and cluster analysis [20,21]. Depressive symptoms associated with burnout are not a response or a transient state but are a constant and prolonged state over time. It seems that they are interconnected, sharing some common characteristics and probably growing together. A relationship between burnout and the different symptoms of depression has been evidenced: anhedonia, disturbed sleep, psychomotor disturbance, fatigue, etc. Longitudinal studies have shown that burnout and depression symptoms are separable—that is, they are intertwined. Thus, individuals who experienced an increase in the states of burnout led to an increase in depression compared to those in whom a concomitant change decreasing in burnout led to less depression. The final stage of burnout is usually associated with high depression—in some cases, with suicidal ideations [20].

However, self-esteem has a relationship between burnout and poor health [22,23,24,25,26,27,28,29]. Thus, people’s self-esteem affects their work, in turn, which means better or worse results [30]. Professional achievement is positively related to self-esteem [24,26]. However, people with low self-esteem have a higher vulnerability; they show higher levels of emotional exhaustion, anxiety, impulsivity compared to people with high self-esteem [27,28,29] and depersonalization and even creating a feeling of incompetence in their relationships with others [26]. In this way, self-esteem affects interpersonal relationships. They tend to show fewer resources to alleviate the stressful effects produced by burnout and are usually vulnerable to environments loaded with greater stressful situations [26,27,28].

It follows from all of the above that teachers who have a high level of burnout may present symptoms of depression. Depression has devastating effects on teachers. However, we must bear in mind that self-esteem can have a protective role against the effects of burnout. Therefore, it is necessary to investigate the profiles of teachers with burnout to detect the levels of symptoms of depression that can be at risk, as well as the levels of self-esteem that can protect the individual.

In a previous study, we analyzed coping strategies in teachers to cope with burnout and depression, finding three profiles of teachers with low, medium and high stress levels. Among the limitations of our previous study, it was necessary to analyze the role of self-esteem in burnout and depression in teachers. Therefore, this study came about [13]. The present study aims to analyze the different burnout profiles and verify if there were differences between burnout profiles in depressive symptomatology and in the self-esteem of the teachers at school.

## 2. Material and Methods

### 2.1. Participants

In total, 20 schools were selected (in the Region of Murcia, Spain), with an average of 15 teachers per institution. The sample was 300 teachers of compulsory secondary education (CSE). Of the 300 subjects who were requested to participate, 216 (72% response rate) answered, and of them, 6 answered less than 50% of the items, so they were removed; the proportion of nonparticipants was similar in each one of the centers, oscillating between 3 and 5. Likewise, 90 (30%) were excluded (had errors or missed answers). The final group reached consisted of 210 teachers: 73.5% public. The age of teachers ranged from 30 to 65 years: under 35 years (27%), 36–55 years (53%) and 55 or more (20%), being 43.8% men. The distribution was homogeneous (see Table 1): by gender and age group (*χ*^2^ = 6.04, *p* = 0.053), gender and professional experience (*χ*^2^ = 3.50, *p* = 0.174) and by gender and administrative category (*χ*^2^ = 6.96, *p* = 0.072). Regarding marital status: 61.9% married, 25.2% single, 3.8% divorced or separated, 6.7% civil union, 1.4% widowed and 1% did not report. Sixty-one point nine percent had more than 10 years of experience. With respect to the working environment, the participating teaching staff taught in secondary schools, mainly with students from 12 to 18 years old, although some schools also had other courses. Working hours were generally in the morning, and the sociocultural level of the environment was average.

### 2.2. Instruments

The MBI (Maslach burnout inventory) [2] was used. It is composed of 22 items, and responses are recorded on a rating scale (from 0 = never to 6 = everyday). The questionnaire has three dimensions: emotional exhaustion EE (e.g., I feel used up at the end of the workday), depersonalization DE (e.g., I worry that this job is hardening me emotionally) and personal accomplishment PA (e.g., I have accomplished many worthwhile things in this job). The Cronbach’s alpha in the original validation study were: EE α = 0.90, DE α = 0.79 and PA α = 0.90 [2]. In this study was EE α = 0.89, DE α = 0.71 and PA α = 0.79.

The second scale was the SDS (self-rating depression scale) by Zung [31,32,33] to measure the behavioral symptoms of depressive disorder. It consists of 20 items, each of which requires responses to be recorded on a rating scale (from 1 = a little of the time to 4 = most of the time). Cronbach’s alpha ranged from 0.79 to 0.92 in several previous studies [32,33]. In the application of this study was α = 0.82. An example of an item from the scale is “I have crying spells or feel like it”.

The RES (Rosenberg self-esteem scale) by Rosenberg [34] is a psychometric instrument composed of 10 items, of which six are positive, to assess the usage of a rate scale (from 1 = strongly agree to 4 = strongly disagree). Scores above those values refer to high self-esteem, while scores below would be related to low self-esteem in the general population. Cronbach’s alpha ranged from 0.77 to 0.88 approximately in several previous studies [7,11,22,24,34]. In this study: negative items α = 0.92 and positive items α = 0.93. Example of item: “At times, I think I am no good at all”.

The sociodemographic variables of the study were the following: gender (male/female), age, type of school (public/private), professional experience (less than 5 years/between 6 and 10 years old/more than 10 years old), administrative category (civil servant/interim/contracted/others) and marital status (married/single/divorced or separated/civil union/widower/did not report).

All the questionnaires were presented in Spanish.

### 2.3. Procedure

Participants were teachers from CSE schools. After obtaining permission, the questionnaires were completed in the educational centers. Questionnaires were filled for about 50 min.

The process was anonymous, voluntary and confidential.

### 2.4. Data Analysis

First, to identify burnout profiles, latent class analysis (LCA) [35] was carried out to classify participants on burnout dimensions (depersonalization, emotional exhaustion and professional fulfillment).

After, analysis of variance (ANOVA) was performed to determine the mean differences between the profiles in SDS and RES using post hoc tests (Bonferroni method). Likewise, the effect size *d* [36] and the Statistical Package for the Social Science version 24.0 (IBM SPSS, Inc., Chicago, IL, USA) and the Excel package (XLSTAT) (Microsoft Corp., Redmont, WA, USA) to run the latent class analyses were used.

### 2.5. Ethics Approval

This manuscript is part of a bigger research. The study protocols were approved by the Ethics Committee (University of Murcia) for Clinical Investigations in September 2019 (ID:2478/2019). This study was performed with written informed consent from all teachers.

## 3. Results

The latent class analysis (LCA) is observed, and the adjustment for each model (two to six classes) is in Table 2. The five-classes model and the six-classes model present few participants and were therefore rejected. For this reason, the three-classes model is chosen, because it has the lowest Bayesian information criterion (BIC) value compared to the two-classes and four-classes. The class solution with the lowest BIC is selected when a model that meets the fit criterion is not available [12,13,37,38]. Hence, the three-class model is selected for having the best fit indices.

**Figure 1:**
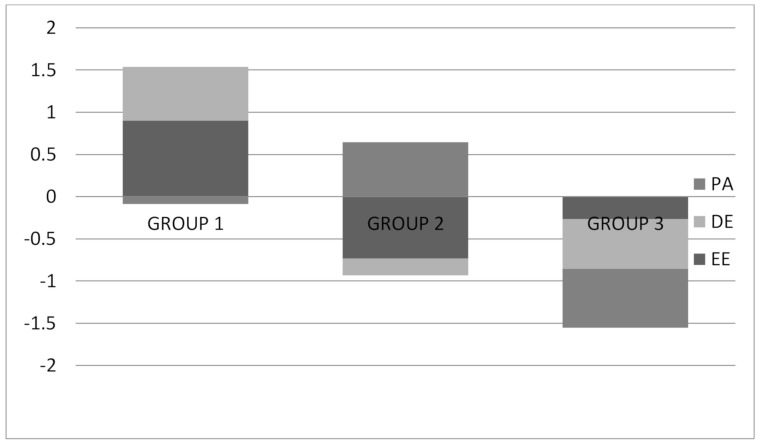
Graphical representation of the model selected. Note: Group 1 (high burnout), Group 2 (low burnout) and Group 3 (moderate burnout). PA: personal accomplishment, DE: depersonalization and EE: emotional exhaustion.

The three emergent burnout profiles are presented in Figure 1. Group 1 consist of 70 teachers (33.3%) with high EE, high DE and low PA; it is called high burnout. The second group consists of 82 teachers (39.1%) with by low EE, low DE and high PA; it is called low burnout. The third group consists of 58 teachers (27.6%) with low DE, low EE and low PA; it is called moderate burnout. Each group represents a different profile. Among the profiles, we found no differences due to the sociodemographic variables of the study.

ANOVAs revealed that there were group differences in relation to self-esteem and depressive symptomatology (see Table 3).

In Table 4, we see the post hoc comparisons between self-esteem and depression in each group. On one hand, Group 2 (low burnout) obtained significantly higher scores on the self-esteem scale than Group 1 (high burnout). However, Group 3 (moderate burnout) did not obtain significant differences with respect to Group 1 (high burnout) and respect to Group 2 (low burnout).

Regarding the depressive symptomatology, Group 1 (high burnout) obtained significantly higher scores than Group 2 (low burnout). Relatedly, Group 3 (moderate burnout) scored significantly higher than Group 2 (low burnout). Finally, Group 1 (high burnout) scored significantly higher than Group 3 (moderate burnout).

## 4. Discussion

Our study was based on investigating the profiles of burnout teachers to detect the levels of depression and their associated symptoms that may be at risk, as well as the levels of self-esteem that can protect the individual. The present study aimed to analyze the different burnout profiles. Through the latent class analysis, three different profiles were identified: the first group of teachers were characterized by high burnout; the second group characterized by low burnout and the third group characterized by moderate burnout. Thirty-three point three percent of teachers have high burnout values, followed by 27.6% of teachers who are at risk for having moderate burnout values. This is in-line with studies that show that teachers are high-risk workers to experience burnout [6,7,8,9,10,11,12,13,14,15,16,17,18,19]. More specifically, it has been found that 18.3% of teachers not only had high levels of burnout but, also, of guilt, not being able to perform their functions optimally and showing feelings of tiredness and frustration [18]. In another study, high levels of emotional burnout and depersonalization were found in 25.9% and 16.9%, respectively [10]. The second aim was to verify if there were significant differences between profiles with respect to depressive symptomatology, as well as self-esteem, among teachers at school. There were significant differences in depressive symptomatology and self-esteem. Regarding the most relevant differences presented by each profile or group, we highlight the most relevant aspects below. Specifically, the results also showed that Group 2 (low burnout) obtained significantly higher scores on the self-esteem scale than Group 1 (high burnout). This implies that teachers in Group 2 are less burnt out. They had high values in professional accomplishments, which are associated with feelings of competence at work and a tendency to evaluate themselves positively, which implies a high self-esteem compared to teachers in Group 1, who are more vulnerable due to having high levels of burnout. In-line with this, several authors have confirmed similar data, since self-esteem has an association between burnout and poor health. Self-esteem allows you to relieve burnout by improving your quality of life [17,18,19,20,21,22,23,24,25]. Thus, people with low self-esteem are more vulnerable to burnout, showing higher levels of EE [28] and depersonalization [26]. Therefore, feelings of competence and effectiveness at work are related to self-esteem, [30] and those with high professional realization values have high self-esteem [26].

In this sense, Group 1 (high burnout) obtained higher scores than Group 2 (low burnout) and Group 3 (moderate burnout) in depressive symptomatology. The teachers in Group 2 were the ones with the lowest level of depressive symptomatology. This is in-line with studies that show that low job satisfaction is related to burnout, lowered self-esteem, anxiety and depression [6], as well as an association between burnout and depression. People who have a high level of burnout usually present depressive symptoms in the final stage of burnout. Therefore, prevention in primary, secondary or tertiary workplaces is necessary [20,21]. In fact, Group 1 has a high level of burnout, and Group 3 is at-risk, because it has moderate burnout values, and low professional achievement values are worrisome, so it is recommended to start management programs for these symptoms [2,17,18,39], which can lead to consequences even in students [17].

Our study is in-line with several studies that have reported an association between burnout and depression in teachers using the MBI test and cluster analysis [20,21]. We have evidenced that the different depressive symptoms are associated with burnout. A relationship has been evidenced between exhaustion and the different symptoms of depression [20].

The results of the study suggest the need to take into account activities or programs that promote self-esteem to reduce burnout and depressive symptomatology, especially in Group 1 (high burnout) and Group 3 (moderate burnout) [23], decrease role ambiguity [18,38,40,41,42,43,44] to promote social support and empathy among teachers [43,45,46,47], develop emotional skills in teachers for the regulation of emotions in stressful situations to reduce the high levels of EE and DE [48,49,50,51,52,53,54], foster illusions at work [55] and to promote the welfare and commitment of individual teachers [24].

Likewise, the results make clear the idea that teacher burnouts should be seen as an organizational problem—since, that way, organizational measures could be applied to address it (i.e., reducing the organizational overload, improving time management, motivating workers, etc.), which will ultimately reduce burnout among teachers and increase productivity and satisfaction in the workplace [45,46].

The fact that the data are self-reported can be pointed out as a limitation of this research study; even social desirability also has to be taken into account in the answers and, also, the study’s cross-sectional design. Due to the relatively small effect of self-esteem obtained in our study, it would be necessary to inquire about the role of self-esteem through interviews or long-term monitoring, as well as the level of depression. Future research could consider: the addiction to new technologies or drug use, history of physical and mental health, medication use [20], teacher’s self-efficacy [50], emotional intelligence [51,52,53,54,55,56,57], meditation [58], resilience [9,12], neurobiological mechanisms behind burnout [21], longitudinal studies [20], tests of mediation [15] and address environmental factors that can influence stress, as well as other variables that may be of interest.

## 5. Conclusions

Burnout is a reality in the teaching profession [6,7,8,9,10,11,12,13,14,15,16,17,18,19]. Therefore, the results obtained corroborate that burnout syndrome is actually present in some secondary school teachers [6,7,8,9,10,11,12,13,14,15,16,17,18,19]. Similar results were found in studies carried out in the Region of Murcia, which were not exclusive to public or subsidized schools [13,59,60]. Three different burnout profiles were found: the first group of teachers was characterized by high burnout; the second group characterized by low burnout and the third group characterized by moderate burnout. Therefore, teachers with certain profiles are workers with a high risk of experiencing burnout [7,8,10,12]. A depressive mood is usually associated with teacher profiles at risk, since they have high EE and DE [20,21]. However, the results showed that self-esteem has a fundamental role in situations of burnout among teachers, since self-esteem can influence the levels of stress or burnout [22,24,25,26,28,29]. As it has been observed in this research, the profile of the teaching staff is associated with different levels of self-esteem, as well as in depressive symptomatology. In particular, those with greater burnout symptoms show higher levels of depression and lower scores in self-esteem (Group 1). The opposite occurs with those teachers who have a high score in professional performance and low in depersonalization and emotional exhaustion (Group 2).

The psychological balance and health of teachers depend on preventing the factors that have been associated with this syndrome [61], which can have consequences even in students [17]. Primary prevention aimed at guaranteeing well-being is essential. It is necessary that all organizations promote the good health of their employees, creating working conditions that promote the welfare of workers, including teachers. It is especially important to improve job satisfaction and motivation [55], performance and the quality of working conditions [44]. In the same way, it is necessary to support people who suffer from a mental problem to reduce absenteeism, increase productivity and obtain the economic benefits that these effects entail [1].

## Figures and Tables

**Table 1:** Sample distribution according to sociodemographic variables and gender.

Variables	Gender	Male	Female
Age group	Under 35 years	22 (10.5%)	36(17.1%)
	36–55 years	48 (22.9%)	62 (29.5%)
	55 years or more	26 (12.4%)	16 (7.6%)
Administrative category	Civil servant	58 (27.6%)	69 (32.9%)
	Interins	17 (8.1%)	13 (6.2%)
	Contracted	15 (7.1%)	30 (14.3%)
	Others	6 (2.9%)	2 (1%)
Professional experience	5 years	20 (9.5%)	26 (12.4%)
	6–10 years	11 (5.2%)	23 (11%)
	10 years or more	65 (31%)	65 (31%)

**Table 2:** Fit indices of the latent profile for all models compared. BIC: Bayesian information criterion.

Account of Classes	BIC	Entropy
2	1754.83	0.63
3	1750.31	0.62
4	1764.09	0.65
5	1784.62	0.69
6	1811.24	0.71

**Table 3:** Means (M) and standard deviations (SD) for the three groups for each measure of self-esteem and depressive symptomatology.

	Group 1		Group 2		Group 3				
Measure	M	SD	M	SD	M	SD	*F_(2,217)_*	*p*	*η_p_^2^*
Self-Esteem	24.39	2.76	25.35	2.38	25.00	1.79	3.86	0.023	0.04
Depressive symptomatology	40.96	8.81	30.18	5.45	34.38	5.94	46.62	<0.001	0.31

Note: Group 1 (high burnout), Group 2 (low burnout) and Group 3 (moderate burnout). ***F*** = F-value; ***p*** = significance; ***ηp2*** = partial eta squared.

**Table 4:** Cohen’s indices for post-hoc contrast groups.

Measures	Group 1-Group 2	Group 1-Group 3	Group 2-Group 3
Self-Esteem	0.18 *	---	---
Depressive symptomatology	1.50 ***	0.86 ***	0.74 **

Note: * *p* < 0.05, ** *p* < 0.01 and *** *p* < 0.01.
